# Neuroprotective Effect of α-Mangostin in Ameliorating Propionic Acid-Induced Experimental Model of Autism in Wistar Rats

**DOI:** 10.3390/brainsci11030288

**Published:** 2021-02-25

**Authors:** Aarti Tiwari, Rishabh Khera, Saloni Rahi, Sidharth Mehan, Hafiz Antar Makeen, Yahya H. Khormi, Muneeb U Rehman, Andleeb Khan

**Affiliations:** 1 Department of Pharmacology, Neuropharmacology Division, ISF College of Pharmacy, Moga, Punjab 142001, India; aartitiwarildh@gmail.com (A.T.); rishabhkhera61199@gmail.com (R.K.); saloninamta@gmail.com (S.R.); 2 Department of Clinical Pharmacy, College of Pharmacy, Jazan University, Jazan 45142, Saudi Arabia; hafiz@jazanu.edu.sa; 3 Division of Neurosurgery, Department of Surgery, Faculty of Medicine, Jazan University, Jazan 45142, Saudi Arabia; yakhormi@jazanu.edu.sa; 4 Department of Clinical Pharmacy, College of Pharmacy, King Saud University, Riyadh 11451, Saudi Arabia; muneebjh@gmail.com; 5 Department of Pharmacology & Toxicology, College of Pharmacy, Jazan University, Jazan 45142, Saudi Arabia

**Keywords:** autism spectrum disorder, ERK/mitogen activated protein kinase (MAPK), α-mangostin, propionic acid, neuroexcitation, genetic dysfunction

## Abstract

Several studies have documented the role of hyper-activation of extracellular signal-regulated kinases (ERK) in Autism pathogenesis. Alpha-mangostin (AMG) is a phytoconstituents with anti-oxidants, anti-inflammatory, and ERK inhibition properties in many diseases. Our research aims to investigate the neuroprotective effect of AMG in the rat model of intracerebroventricular-propionic acid (ICV-PPA) induced autism with a confirmation of its effect on the ERK signaling. Autism was induced in Wistar rats (total 36 rats; 18 male/18 female) by multiple doses of PPA through ICV injection for 11 days. Actophotometer and beam walking tasks were used to evaluate animals’ motor abilities, and the Morris water maze task was utilized to confirm the cognition and memory in animals. Long term administration of AMG100 mg/kg and AMG200 mg/kg continued from day 12 to day 44 of the experiment. Before that, animals were sacrificed, brains isolated, morphological, gross pathological studies were performed, and neurochemical analysis was performed in the brain homogenates. Cellular and molecular markers, including ERK, myelin basic protein, apoptotic markers including caspase-3, Bax, Bcl-2, neuroinflammatory markers, neurotransmitters, and oxidative stress markers, have been tested throughout the brain. Thus, AMG reduces the overactivation of the ERK signaling and also restored autism-like behavioral and neurochemical alterations.

## 1. Introduction

Autism is a complex neurodevelopmental condition that indicates language disability, repetitive and irregular social interaction movements, sensory disturbances, hyperactivity, and occasionally symptoms of self-injury [1]. Multiplestudies have shown that different factors such as heavy metals (Mercury) [2], valproic acid and thalidomide [3], cosmetics and perfumes [4], environmental toxicity [5], gene mutation [6], and intestinal dysbiosis [7] contribute to the pathogenesis of autism. ASD (autism spectrum disorder) affects 1–2 percent of the population and is five percent more common in adult males than females. Approximately 10 million children who are vulnerable to autism, i.e., 1–1.5% or 1 out of 66 children under the age of 2–9, are affected in India [8].

Usually, animal models are used to assess the disease’s pathological mechanisms and propose potential treatments for biological functions. Curiously, findings show that ICV-PPA infusion can induce neurobehavioral abnormalities in rodents similar to those seen in autism patients by altering fatty acid metabolism [9]. PPA is imported for normal and immune physiological level related functions, but elevated levels may alter the immune and metabolic processes and worsen the stereotyping behaviors associated with ASD. PPA tends to affect the functioning of the central nervous system, including gap junction communication, neurotransmitter synthesis and release, immune activation and mitochondrial function, lipid metabolism, and gene expression [10].

PPA is capable of developing ASD-like syndrome when administered via ICV and induces behavioral dysfunction [11], neurotransmitter imbalance [9], which impairs the cognitive and memory of rodents. PPA also interferes with the neuroinflammatory response, oxidative stress [12] and alters the mitogen-activated protein kinase (MAPK) signaling pathways [13], and increases the level of the extracellular signal-regulated kinase (ERK) [14]. The ICV-PPA induced animal models of autism are now a widely used pre-clinical model for autistic therapy evaluation. These models fully meet all criteria and resemble behavioral and neurochemical alterations similar to people with ASD and are easily established and evaluated in laboratory conditions.

MAPK protein kinases transform extracellular stimuli via a wide range of cellular responses. MAPKs are one of the earliest signaling pathways and are widely used to develop many biological processes [15]. ERK1/2 can play an essential role in the development of the neuron and activation of the adult brain. In adult nervous systemregulation, the adult nervous systemregulation, the adult nervous systemregulation, the adult nervous system regulation, ERK1 and ERK2 are closely linked to neuroinflammation, neuronal death, memory development, and learning and synaptic plasticity [16,17,18]. Synaptic plasticity is considered essential for processing brain information and underlies several complex behaviors; regulating protein phosphorylation plays a significant role in both long-term potentiation (LTP) and long-term depression (LTD). ERK1/2 is found in the soma and dendritic neurons clusters of the neocortex, hippocampus, striatum, and cerebellum. ERK1/2 is needed for the formation of memory and learning; it is also found in oligodendrocytes, astrocytes, and microglia, where it controls the production of cytokines and neuroinflammation [19,20]. 

Aberrant ERK signaling is symptomatic of ASD-associated neurological disorder pathogenesis, a mutation of the component of these pathways results in a synaptic plastic defect in the syndromic type of autism that the causative gene product interferes with local protein synthesis [21,22]. ERK signaling dysfunction is related to a variety of neurological abnormalities such as Huntington’s disease (HD), Parkinson’s disease (PD) [16], Alzheimer’s disease (AD) [23], amyotrophic lateral sclerosis (ALS), multiple sclerosis (MS), stroke, ischemia [24], asthma [25] and cancer [26]. The modulation of elevated ERK signaling pathways by multiple ERK inhibitors has demonstrated promising results in the treatment of various diseases such as AD [27], brain injuries [28], PD [29], HD [30] autoimmune disease [31], and ASD [32].

AMG is one of the main xanthonesisolated from pericarp berries, dried sap, and mangosteen tree barks (*Garcinia mangostana*) also known as “the queen of berries.” AMG has a range of pharmacological interventions, including anti-inflammatory [33,34] anti-oxidants [35], antibiotics [36], and neuroprotective activities [37]. Its bioavailability is relatively narrow due to the hydrophobic characteristics of AMG [38]. AMG exhibits antagonistic effects on ERK pathways and inhibits the elevated level of ERK in ASD [39,40,41]. Several studies have shown that decreased serotonin levels [42], glial cell activation [43], oxidative stress production [44], and neuroinflammation [45] play a role in autism pathogenesis.

AMG inhibits acetylcholinesterase (AChE) [46] and butyrylcholinesterase (BChE) and improves the function of serotonin [47]. AMG also blocks the tumor necrosis factor (TNF-α), interleukin (IL-β), and inhibits the process of neuroinflammation by reduction of inflammatory cells infiltration and generation of cytokines from Kupffer cells. It also increased CYP2E1 and GPX gene expression which decreased TNF-α concentration [48]. Several studies indicate the neuroprotective effect of AMG in different diseases such as AD [49], HD [50], PD [51], cognitive disability [52], schizophrenia [53], depression [54], andcancer [55].

The suppression of ERK signaling by AMG may have the ability to reduce and delay the progression of autism-related neuro-abnormalities and can act as preventive ASD treatment [56,57]. Therefore, the present research establishes the ERK/MAPK signaling mechanism for autism pathogenesis and examines the neuroprotective effect of AMG on behavioral, neurochemical, and morphological parameters in the animal autism model.

## 2. Materials and Methods

### 2.1. Experimental Animals

A total of 36 animals (adult Wistar rats) weighing 180 to 220 g (18 male/18 female) were used in the protocol. Rats were purchased from ISF College of Pharmacy, Moga, Punjab, India, from the Central Animal House. For at least one week before the protocol starts, all animals have been maintained under the 12-h dark cycle of light/12 h. Free access to food and water for all laboratory animals. Poly-acrylic cages with at-least three animals per cages were used during the protocol. All behavioral parameters were examined between 9 a.m. and 5 p.m. The study protocol was approved with protocol number ISFCP/IAEC/CPCSEA/Meeting No.25/2019/Protocol No.409 by the Committee on Institutional Animal Ethics (816/PO/ReBiBt/S/04/CPCSEA). The trials were performed in conformity with the Indian National Science Academy guidelines on the use and care of experimental animals (INSA).

### 2.2. Chemicals and Drugs

Propionic acid (PPA) was purchased from Sigma–Aldrich (USA). AMG was provided an *ex-gratia* sample from BAPEX, India. All other chemical substance used in the study is analytical grades. Drugs and chemical solutions are freshly prepared before use. AMG dissolved in dimethyl sulfoxide (DMSO) and administered through oral route (*p.o*) [58]. 

### 2.3. Experimental Protocol Schedule 

The total duration of the study was 44 days. The PPA-induced experimental model of autism in the rat was conducted according to the method established by Sharma et al., 2019 [12]. On day 1st propionic acid (PPA) was administered intracerebroventricularly (ICV). ICV-PPA is chronically injected from day 1st to day 11th. AMG chronically administered from day 12th to end of study (day 44th) (Figure 3). Animals were randomly assigned to six groups. Group 1 Vehicle control, Group 2 Sham control, Group 3 AMG200 *per se*, Group 4 PPA, Group 5 PPA + AMG100, Group 6 PPA + AMG200. During protocol days, behavioral parameters such as beam crossing, locomotion, forced swimming, and morris water task were carried out. Animals were carefully anesthetized with (270 mg/mL, i.p.) pentobarbital sodium and transcardially perfused with (0.1 M) PBS on day 45, followed by PBS paraformaldehyde (4%) for biochemical brain analysis. 

### 2.4. Experimental Model of Autism

**Figure 1:**
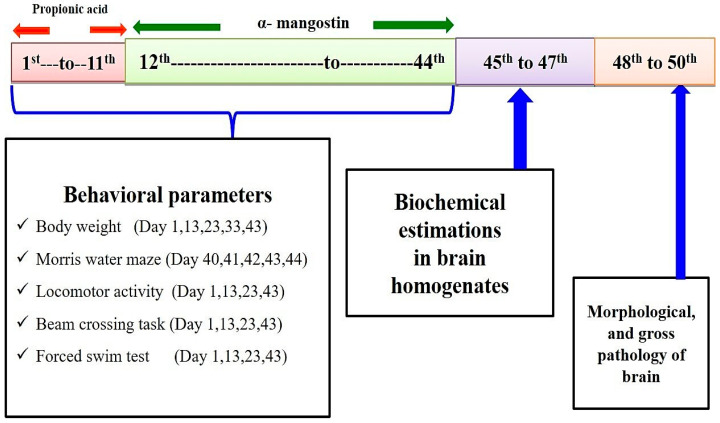
Experimental protocol schedule (Behavioral and biochemical estimations).

Ketamine (75 mg/kg, i.p.) was used to anesthetize laboratory rats and was put in a stereotactic surgical setting shortly afterward. The PPA-induced experimental model of autism in the rat was conducted according to the method established by Sharma et al., 2019 [12]. The head was shaved, and the skull was exposed to a midline scalp incision. A burr hole was formed to be inserted into the cerebroventricular portion of the brain with a calibrated Hamilton syringe by following coordinates with reference to Bregma: −1.4 mm anterior/posterior (AP); 1.8 mm medial/lateral (ML); −3.0 mm dorsal/ventral (DV). After completing the injection, the burr hole was filled with dental cement and fixative, and the skin was sutured with a surgical thread. After surgery, all rats were treated with twice-daily injections of subcutaneous analgesic (ketoprofen, 1 mL/kg) and antibiotic gentamycin (35 mg/kg, i.p.) twice daily in 12 h to avoid infections. After surgery, oral glucose and regular chow diets were fed to animals for four days. Animals are then supplemented by a standard diet of water and chow. The entire experimental study and protocol are described in Figure 1.

### 2.5. Parameters Assessed

#### Measurement of Body Weight

The body weight was assessed on days 1st, day 13th, 23rd, 33rd, and 43rd of the experiment [59].

### 2.6. Behavioral Parameters

#### 2.6.1. Spontaneous Locomotor Activity

On days 1st, 13th, 23rd, 43rd, the locomotion was monitored by actophotometer (INCO (instruments and chemicals private limited), Haryana). Motor activity detected in the device through photocells. Before the recording, the animals were individually placed in the activity room for three minutes forhabituation. Over five minutes, each animal was seen and shown as a count for five minutes [60].

#### 2.6.2. Morris Water Maze Task

The Morris Water Maze (MWM) was used to test escapelatency on days 40th, 41st, 42nd, 43rd, and the time spent in the target quadrant (TSTQ) measured on days 44 to evaluate cognitive function. As with the memory test in the southwest quadrant, a platform was placed 2 cm underwater. A total number of four trials per day were taken. Before day 0, every animal was trained in different directions, starting from one release point. There was a particular gap of 15 min in each trial. The working memory was executed 30 s after hemorrhage induction on the day, and escape latency was also measured. When 10 s or 120 s had elapsed for the platformstationed rat, each reference and work memory test was completed. In 120 s, when the rats were not found, they were directed to stand on the platform, and then the latency of the escapewas recorded in 120 s. On day 44, rats were tested, and a TSTQ assessment was carried out, and the platform was removed [61].

#### 2.6.3. Beam Crossing Task

On days 1st, 13th, 23rd, and 43rd days, the motor co-ordination ability of each animal were tested. The slip numbers were recorded in each study and the motor performance of rats was also measured on a scale of 0–4. An animal that could easily cross the beam was assigned a score of 0. Animals with mild, moderate, and severe disabilities were given scores 1, 2, and 3, respectively. The fourth score was given to the animals that could not go on the beam [62].

#### 2.6.4. Force Swim Test

On day 1st, 13th, 23rd, and 43rd tests have been performed to assess the depressive performance of rats. The first exposure in the tank for rats takes 15 min during the training phase, while the second is done 24 h after the first exposure, with a 5 min exposure time. The test period was the 6-min exposure for rats, the first 2 min are a habit period and the last 4 min are a test itself which results in the duration of the immobility [63].

### 2.7. Measurement of Cellular and Biochemical Markers

#### 2.7.1. Brain Homogenate Preparation

On day 45, decapitation of animals was performed. Brains have been purged and washed with an isotonic solution of ice-cold saline. A 0.1 M phosphate buffer with a chilled phosphate buffer (7.4) was then homogenized brain sections. The homogenous substances were then centrifuged at 10,500× *g* for the next 15 min, and the supernatant separated [64].

#### 2.7.2. Measurement of ERK Levels

An ELISA kit (Elabsciences, China) was used to measure ERK levels in rat’s brain homogenate supernatant, and the values were expressed in percentages [65].

#### 2.7.3. Measurement of Myelin Basic Protein (MBP)

Rat myelin basic protein level is measured using ELISA kit from Elabsciences, China in rat’s brain homogenate supernatant, and the values were expressed in μg/mg total protein [66].

### 2.8. Measurement of Apoptotic Markers

#### 2.8.1. Caspase-3 Levels

Caspase-3 levels were measured by ELISA kit (Elabsciences, China) in the brain homogenate. The procedure was performed according to the instructions of the manufacturer. The assay employs the enzyme immunoassay competitive method [67].

#### 2.8.2. Bax and Bcl-2 Levels

ELISA kit was used to measure both Bcl-2 protein and Bax protein levels (Elabsciences, China) in the brain homogenate. The method was followed according to the instructions of the manufacturer. Bax and Bcl-2 levels expressed in ng/mg protein [68,69].

### 2.9. Neurotransmitters Evaluation

#### 2.9.1. Serotonin Levels

HPLC (high-performanceliquid chromatography) with an electrochemical detector was evaluated for serotonin levels in rat brain homogenate. The mobile phase included buffer (sodium citrate), a ratio of 87:13 *v*/*v* (pH 4.5), and flux of one mL/min for smooth separation, whereas ng/mg protein was recorded. The levels of serotonin as ng/mg protein have been reported [70].

#### 2.9.2. Glutamate Levels

Glutamate levels were assessed after derivatization with o-phthalaldehyde/β-mercaptoethanol(OPA/β-ME) in the tissue sample, and data were represented as mg/mg protein [71].

#### 2.9.3. Dopamine Levels

To estimate brain dopamine levels, high-performance liquid chromatography (HPLC) was used. Before being centrifuged, the brain samples have been thawed and homogenized with 0.2 M perchloric acid. Before the injection into the sample injector with 0.22 mm nylon filters, the supernatant was purified. Data collection and analysis were carried out using Breeze software. Electrochemical detector sensitivity (ECD) ranged from 5 to 50 nA. data was represented in mg/mg protein [72].

#### 2.9.4. Acetylcholine (Ach) Levels

To measure the acetylcholine, an ELISA kit (ELab Sciences, Wuhan, China) was used. As specified in the kit, all the samples and reagents were prepared. The microtitre plate optic density for the reaction mixture was estimated to be 540 nm. Data was presented in ng/mg protein [73].

### 2.10. Evaluation of Neuroinflammatory Biomarkers

#### TNF-α and IL-1β Levels

The ELISA kits were used to analyze the inflammatory cytokines such as TNF-α and IL-1β (ELab Sciences, Wuhan, China). Samples and reagents were prepared According to the manufacturing protocols of the kit. Mixture optical density was determined at 450 nm on the microtiter plate. Data was presented in pg/mg protein [74,75].

### 2.11. Evaluation of Oxidative Stress Parameters

#### 2.11.1. Lactate Dehydrogenase (LDH) Levels

For the estimation of the LDH, the UV-spectrophotometric method was used. The oxidation of lactate to pyruvate was carried out through LDH. The NAD (nicotinamide adenine dinucleotide, oxidized form) has been reduced to NADH (nicotinamide adenine dinucleotide, reduced form). Serum LDH activity is proportional to the increase in absorption because NAD is decreased. LDH activity was measured using an LDH kit in the rat brain homogeneous (Transasia Bio-Medicals Ltd., Mumbai, India), and it was expressed as IU/L [76].

#### 2.11.2. Acetylcholinesterase (AChE) Levels

Ellman’s method, 1961, as described, quantitatively measured acetylcholinesterase activity. The mixture of the test’s composition consisted of supernatant—0.5 mL, sodium phosphate 0.01 M (pH 8)—3 mL, acetylthiocholine iodide—0.10 mL, and DTNB—0.10 mL. The supernatant’s enzymatic activity was expressed as μM/mg [77].

#### 2.11.3. Glutathione Levels

The glutathione levels were mentioned in total brain homogenate with 1 mL of supernatant digested at 4 °C at approximately 1 h at 1 mL of 4% sulfosalicylic acid and cold digestion. The samples were then centrifuged for about 15 min at 1200× *g*. In addition to 1 mL of the supernatant, 2.7 mL of a phosphate buffer (0.1 M, pH 8) was added, and 1 mL of 5,5′-dithiobis-(2-nitrobenzoic acid)(DTNB) were added. With a Shimadzu-UV spectrophotometer, immediately at 412 nm, the developed yellow color was measured. The levels of glutathione are represented in μM/mg protein [78].

#### 2.11.4. Malondialdehyde (MDA) Levels

Quantitative measurements were taken for the lipid peroxidation in the brain homogenates asend product malondialdehyde (MDA). After its thiobarbituric acid reaction, the MDA was measured with a Shimadzu-UV spectrophotometer at 532 nm. The MDA protein concentration was indicated as nM/mg protein [79].

#### 2.11.5. Superoxide Dismutase (SOD) Levels

The activity of the SOD was assessed by the testing system that used the composed of EDTA (0.1 mmol/L), sodium carbonate (50 mmol/L), and nitro blue tetrazolium (96 mmol/L). The cuvette added 2 mL of the mixture as mentioned above, 0.05 mL of hydroxylamine, and 0.05 mL of supernatant. Auto-oxidation was measured at 560 nmat a 30-s interval at two min with hydroxylamine measurements (Shimadzu-UV, USA). SOD activity was presented in units/mg of protein [80].

#### 2.11.6. Nitrite Levels

Accumulation of nitrite in the supernatant indicated by a colorimeter Greiss reagent test (0.1 percent N-(1-naphthyl) and ethylenediamine 1 percent dihydrated chloride and phosphoric acid 2,5 percent) as an indicator of production of nitric oxide (NO). About the same volumes have been taken and mixed by the supernatant and Greiss reagent. The mixture was then incubatedin the dark for approximately 10 min, and spectrophotometrically absorption was determined at 540 nm. A standard sodium nitrite curve was calculated as the nitrite concentration of supernatant as μg/mL protein [81].

### 2.12. Gross Pathological Examination and Morphology

On day 45, animals were decapitated; for gross pathologic analysis, the brains were removed from the body. After examining the entire rat brain, the coronal sections have been taken for further assessment [82,83]. The 2 mm thickbrain section has been placed on glass slides (coronally from the anterior to the cerebral cortex’s posterior).To perceive all brain areas that encompass the entire striatum, a digital camera (Sony A7 III digital camera, Japan) was used. The digital images of all brain areas, including hematoma, took less than five minutes on each part of the brain. The captured digital images have been filtered for illustration purposes. They were converted into TIFFs (tagged image file format). After completing the procedure, the hematoma region (mm) in each brain section was measured using the image MOTICAM-BA310 plus 2.0 analysis software. The volume of the demyelination scale (mm) was calculated for each coronal brain segment by conversion of the demyelination region (mm) [84]. The demyelination size (mm^3^) in each brain section was determined from the dark greyish area near the striatum by image analysis on the 45th day. The injury’s size was calculated in each coronal 2-mm-thick brain section by calculating the demyelination area (l × b × h) [85,86].

### 2.13. Statistical Analysis

All results have been expressed as the mean and standard error of the mean (SEM). Data were analyzed using two-way ANOVA followed by post-hoc Bonferroni and one-way ANOVA repeated measurements followed by post-hoc Tukey’s Multi Comparison Test. *p* < 0.05 was marked as statistically significant. Data was found to be normalized, and the sample size was not calculated prior.

## 3. Results

### 3.1. Effect of AMG on Body Weight of PPA-Treated Autistic Rats

**Figure 2:**
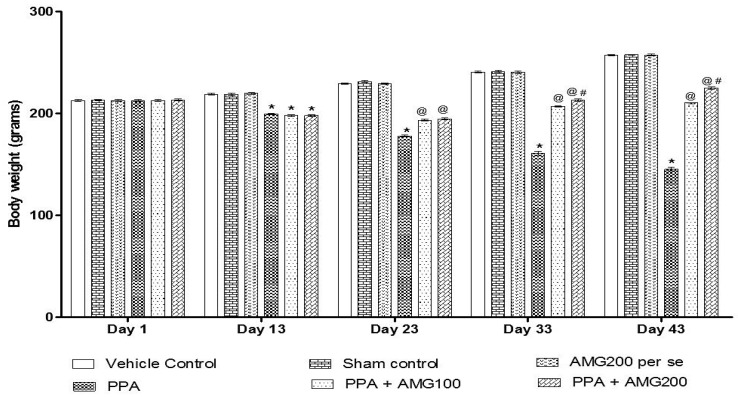
Effect of alpha-mangostin (AMG) on body weight in propionic acid (PPA)-treated autistic rats. Statistical analysis followed by two-way ANOVA (post-hoc Bonferroni’s test), * *p* < 0.0001 v/s vehicle control; sham control and AMG200 *per se*; @ *p* < 0.0001 v/s PPA; @# *p* < 0.0001 v/s PPA + AMG100; (*n* = 6 rats per group).

The body weight was measured on the first, 13th, 23rd, 33rd, and 43rd days of the protocol schedule. On day 1 there is no substantial difference between all groups. PPA-induced rats showed a steady decline in body weight throughout the administration. A significant reduction in the bodyweight of the PPA group was conducted at the end of the experimental protocol (*p* < 0.0001). Long-term therapy with 100 mg/kg AMG and 200 mg/kg AMG on days 23 and 33 restored body weight loss relative to the group treated with PPA [two-way ANOVA: F(20,120) = 551.3; *p* < 0.0001]. AMG200 mg/kg proved to be more significant on days 33 and 43 of the protocol schedule (Figure 2).

### 3.2. Behavior Parameters

#### 3.2.1. Effect of AMG on Locomotion Activity in PPA-Treated Autistic Rats

**Figure 3:**
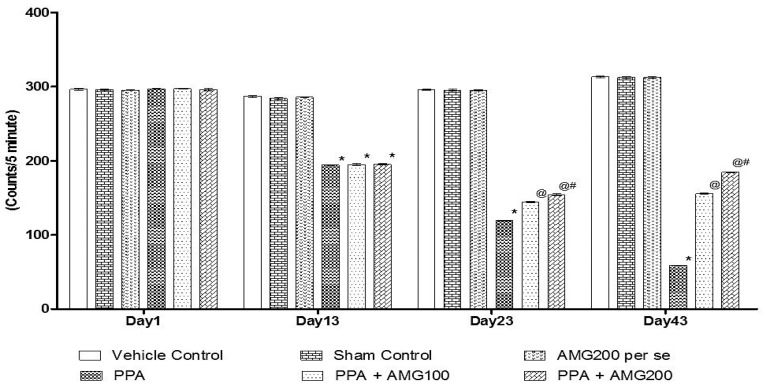
Effect of AMG on locomotor activity in PPA-treated autistic rats.Statistical analysis followed by two-way ANOVA (post-hoc Bonferroni’s test), * *p* < 0.0001 v/s vehicle control; sham control and AMG200 *per se*; @ *p* < 0.0001 v/s PPA; @# *p* < 0.0001 v/s PPA + AMG100; (*n* = 6 rats per group).

Tlocomotive’s behavior was observed by the protocol schedule using the actophotometer on days 1, 13, 23, and 43. On Day 1 there is no substantial difference between all groups. On the 13th day, PPA-induced rats displayed a significant decrease in locomotivemovements relative to the sham group and the AMG200 *per se* group (*p* < 0.0001). Chronic administration of AMG100 mg/kg and AMG200 mg/kg significantly and dose-dependently on day 23 and 43 increased locomotive activity compared to the PPA treated group [two-way ANOVA: F(15,90) = 2142; *p* < 0.0001]. Among these, AMG200 mg/kg was more persuasive in increasing locomotive behavior on day 43 (Figure 3).

#### 3.2.2. Effect of AMG on Spatial Memory in PPA-Treated Autistic Rats

**Figure 4:**
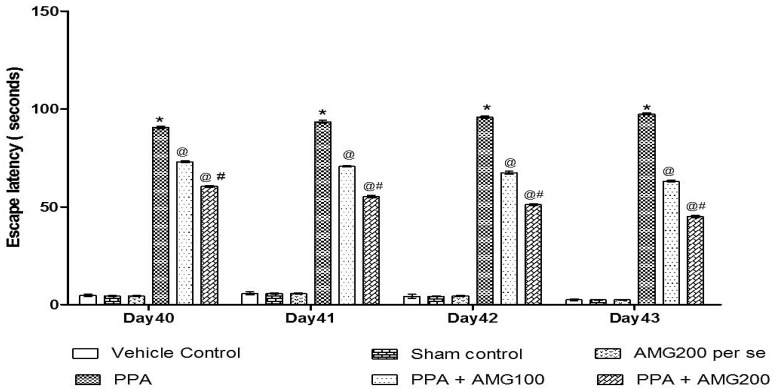
Effect of AMG on escape latency in PPA-treated autistic rats. Statistical analysis followed by two-way ANOVA (post-hoc Bonferroni’s test), * *p* < 0.0001 v/s vehicle control; sham control and AMG200 *per se*; @ *p* < 0.0001 v/s PPA; @# *p* < 0.0001 v/s PPA + AMG100; (*n* = 6 rats per group).

**Figure 5:**
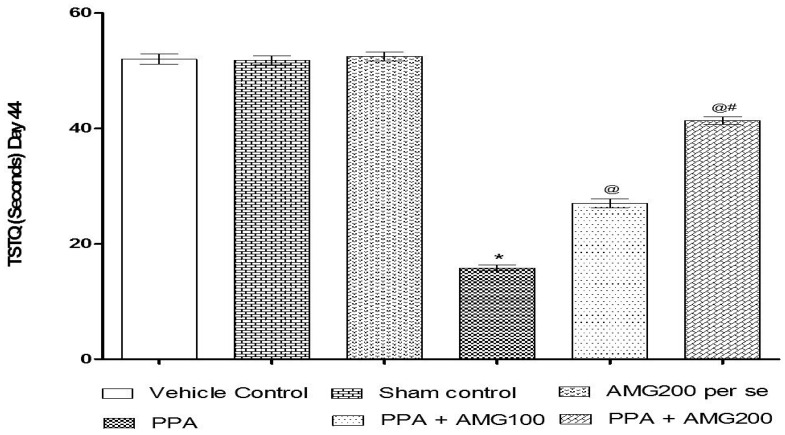
Effect of AMG on time spent in the target quadrant (TSTQ) in PPA-treated autistic rats. Statistical analysis followed by two-way ANOVA (post-hoc Bonferroni’s test), * *p* < 0.0001 v/s vehicle control; sham control and AMG200 *per se*; @ *p* < 0.0001 v/s PPA; @# *p* < 0.0001 v/s PPA + AMG100; (*n* = 6 rats per group).

The ELT was assessed on days 40, 41, 42, and 43, and the TSTQ was assessed on day 44. Based on the findings, a progressive increase in ELT and decreased TSTQ time was observed in PPA-treated rats. A significant decrease in TSTQ and a rise in ELT (*p* < 0.0001) on the 40th day and continues to decrease on the 41st, 42nd, 43rd, and 44th days. Chronic administration of AMG100 mg/kg and AMG200 mg/kg dose-dependent and substantially restored ELT [two-way ANOVA: F(15,90) = 51.72; *p* < 0.0001] and TSTQ [one-way ANOVA: F(15,90) = 1.663; *p* = 0.180] relative to the PPA treatment group. Efficient restoration in the AMG200 mg/kg group showing long-term memory improvement (Figure 4 and Figure 5).

#### 3.2.3. Effect of AMG on Muscle Coordination in PPA-Treated Autistic Rats

**Figure 6:**
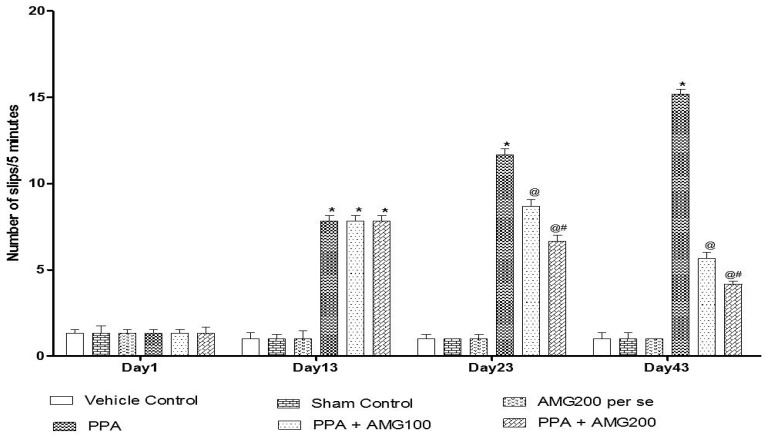
Effect of AMG on muscle coordination in PPA-treated autistic rats.Statistical analysis followed by two-way ANOVA (post-hoc Bonferroni’s test), * *p* < 0.0001 v/s vehicle control; sham control and AMG200 *per se*; @ *p* < 0.0001 v/s PPA; @# *p* < 0.0001 v/s PPA + AMG100; (*n* = 6 rats per group).

The beam crossing task was performed on days 1, 13, 23, and 43 to observe the muscles’ coordination. No major difference between all treatment groups was observed on Day 1. On day 13, the number of slips treated with PPA rats increased significantly (*p* < 0.0001), and on day 23 and 43, chronic AMG100 mg/kg and AMG200 mg/kg dose-dependent administration reduced the number of slips relative to the PPA treatment group [two-way ANOVA: F(15,90) = 75.87; *p* < 0.0001]. Whereas AMG200 mg/kg reduced slip count and improved beam efficiency (Figure 6).

#### 3.2.4. Effect of AMG on the Immobility Phase of PPA-Treated Autistic Rats

**Figure 7:**
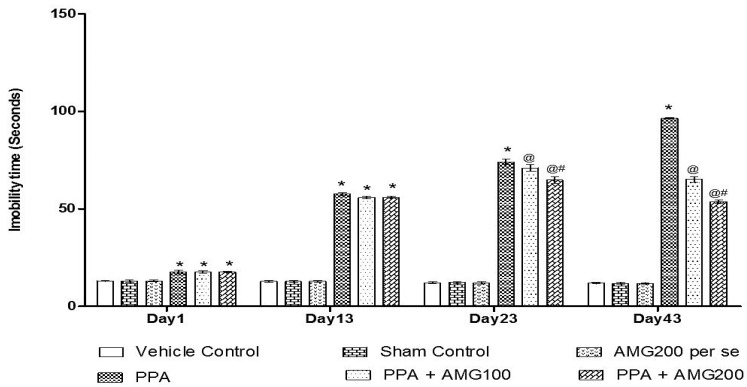
Effect of AMG on immobility phase in PPA-treated autistic rats. Statistical analysis followed by two-way ANOVA (post-hoc Bonferroni’s test), * *p* < 0.0001 v/s vehicle control; sham control and AMG200 *per se*; @ *p* < 0.0001 v/s PPA; @# *p* < 0.0001 v/s PPA + AMG100; (*n* = 6 rats per group).

Under the protocol schedule, the time of immobility was observed using a forced swim test on days 1, 13, 33, and 43. No significant difference between all treatment groups was observed on Day 1. Chronic PPA administration in rats showed a substantial improvement in immobility time (*p* < 0.0001) at the end of the phase. A large increase in immobility time found on the 13th day of the treatment schedule, on the 23rd day and the 43rd day chronic AMG100 mg/kg and dose-dependent AMG200 mg/kg reduces immobility relative to the PPA treatment group [two-way ANOVA: F(15,90) = 376.3; *p* < 0.0001]. Whereas AMG200 mg/kg recovered depressive behavior (Figure 7).

### 3.3. Neurochemical Parameters

#### 3.3.1. Effect of AMG onERK Level in PPA-Treated Autistic Rats

In order to test the inhibitory effect of AMG on PPA-induced autistic animals, we estimated the level of ERK in brain homogenate at the end of the protocol schedule using ELISA kits. PPA-treated rats led to a significant increase compared to the vehicle, sham, and AMG200 *per se* group. Chronic administration of AMG100 mg/kg and AMG200 mg/kg resulted in normalization of the ERK level relative to the PPA treated groups [one-way ANOVA: F(5,25) = 2.947; *p* = 0.030] (Table 1).

#### 3.3.2. Effect of AMG on Myelin Basic Protein Levels in PPA-Treated Autistic Rats

To test the protective action of AMG on PPA-induced autistic animals, we estimated the amount of MBP in brain homogenate at the end of the protocol schedule with ELISA kits. The PPA group resulted in a large increase relative to the vehicle, sham, and AMG200 *per se* group. Chronic administration of AMG100 mg/kg and AMG200 mg/kg resulted in restoration of the MBPlevel relative to the PPA treated groups [one-way ANOVA: F(5,25) = 0.285; *p* = 0.916] (Table 1).

### 3.4. Effect of AMG on the Apoptotic Marker of PPA-Treated Autistic Rats

#### Effect of AMG on Caspase-3, Bax, and Bcl-2 Levels in PPA-Treated Autistic Rats

Caspase-3, Bax, and Bcl-2 were tested in the ratbrain homogenateat the end of the protocol schedule. PPA-treated rats resulted in a significant increase in caspase-3 and Bax levels, while Bcl-2 levels decreased compared to the vehicle, sham, and AMG200 *per se* group. Prolonged administration of AMG100 mg/kg and AMG200 mg/kg resulted in a significant decrease in caspase-3 levels [one-way ANOVA: F(5,25) = 0.936; *p* = 0.474] and Bax [one-way ANOVA: F(5,25) = 0.611; *p* = 0.692] levels and increased Bcl-2 levels [one-way ANOVA: F(5,25) = 0.833; *p* = 0.538] relative to the PPA groups (Table 2).

### 3.5. Effect of AMG on the Measurement of Neurotransmitters in PPA-Treated Autistic Rats

Neurotransmitters such as serotonin, glutamate, dopamine, acetylcholine were assessed in brain homogenates at this end of the experiment protocol. PPA-induced rats resulted in a substantial decrease in serotonin, dopamine, acetylcholine, and an increase in glutamate concentration compared to the vehicle, sham, and AMG200 *per se* group. Persistent treatment with AMG100 mg/kg and AMG200 mg/kg resulted in a substantial rise in serotonin [one-way ANOVA: F(5,25) = 48.76; *p* < 0.0001], dopamine [one-way ANOVA: F(5,25) = 0.484; *p* = 0.784], and Ach [one-way ANOVA: F(5,25) =1.933; *p* = 0.124] concentration and decrease in glutamate [one-way ANOVA: F(5,25) = 0.162; *p* = 0.974] concentration compared to the PPA-treated groups (Table 3).

### 3.6. Effect of AMG on the Assessment of Inflammatory Cytokines in PPA-Treated Autistic Rats

Inflammatory cytokines, such as TNF-α and IL-1β, were analyzed in the rat brain homogenate at this end of the experiment protocol. PA group resulted in a substantial rise in both inflammatory cytokines (TNF-5-007 and IL-1β) relative to the vehicle, sham, and AMG200 *per se* group. Long-term administration of AMG100 mg/kg and AMG200 mg/kg led to a significantreductionof inflammatory cytokines (TNF-α [one-way ANOVA: F(5,25) = 0.573; *p* = 0.719] and IL-1β [one-way ANOVA: F(5,25) = 1.617; *p* = 0.192]) relative to PPA treated classes. (Table 4).

### 3.7. Effect of AMG on the Measurement of Oxidative Stress Markers in PPA-Treated Autistic Rats

At the end of the procedure schedule, oxidative stress markers including AchE, LDH, MDA, Nitrite, GSH, and SOD were evaluated in the brain homogenate. The PPA group resulted in a significant increase in the levels of AchE, LDH, MDA, and Nitrite and a significant decrease in the levels of GSH and SOD compared to the vehicle, sham, and AMG200 *per se* group. Long-termadministration of AMG100 mg/kg and AMG200 mg/kg resulted in a substantial reduction and restoration of AchE [One-way ANOVA: F(5,25) = 3.248; *p* = 0.021], LDH [One-way ANOVA: F(5,25) = 3.924; *p* = 0.020], MDA [One-way ANOVA: F(5,25) = 1.786; *p* = 0.152], and Nitrite [One-way ANOVA: F(5,25) = 0.530; *p* = 0.751] and increase of GSH [One-way ANOVA: F(5,25) = 2.350; *p* = 0.070] and SOD [one-way ANOVA: F(5,25) = 2.909; *p* = 0.033] as compared to ICV-PPA treated group (Table 5).

### 3.8. Effect of AMG on Whole Rat Brain and Brain Sections in PPA-Treated Autistic Rats

#### 3.8.1. Assessment of the Whole Brain in PPA-Treated Autistic Rats

**Figure 8:**
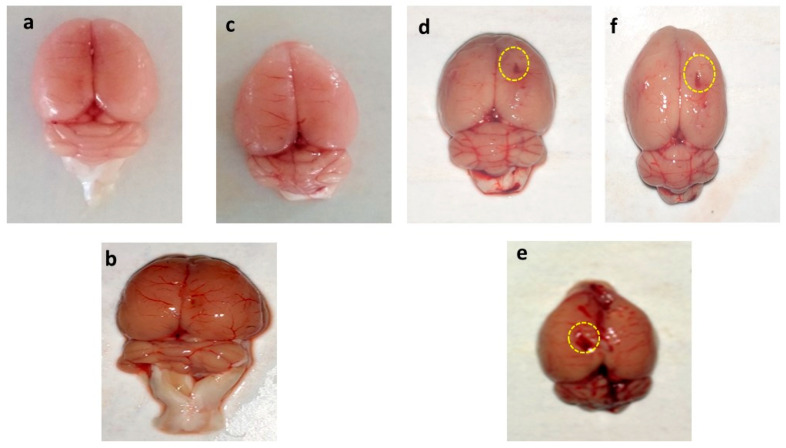
Effect of AMG on gross pathological changes (whole rat brain) in PPA-treated autistic rats. (**a**) Vehicle control (**b**) Sham control (**c**) AMG200 *per se* (**d**) PPA (**e**) PPA + AMG100 (**f**) PPA + AMG200. *(Scale bar= 2 mm). Note: Yellow circles are showing site of injury.*

The whole rat brain of PPA-treated rats was accompanied by weakened and damaged meninges compared to AMG200 mg/kg *per se*, sham, and vehicle-treated rats. The brains of the vehicle, sham, and pers grouped rats were optimally sized with clearly visible meninges. Long-term treatment with AMG200 mg/kg *per se* did not affect whole morphological features thanvehicle and sham-treated rats. morphological improvement was significantly observed following administration of AMG100 and 200 mg/kg (Figure 8).

#### 3.8.2. Assessment of Brain Sections in PPA-Treated Rats

**Figure 9:**
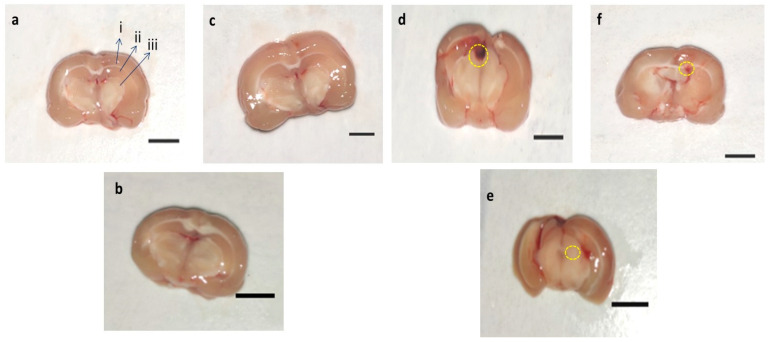
Effect of AMG on gross pathological changes (brain section in PPA-treated autistic rats. (**a**) Vehicle control i. Cerebral cortex ii. Hippocampus iii. Basal ganglia (**b**) Sham control (**c**) AMG200 *per se* (**d**) PPA (**e**) PPA + AMG100 (**f**) PPA + AMG200. *(Scale bar = 5 mm). Note: Yellow circles are showing site of tissue damage.*

In-vehicle control, sham control, and AMG200 *per se* group clear brain tissue were observed during coronal sectioning. The coronal sectioning of the PPA treated brain of the rat showed swelling, cortical contusion, hippocampus damage, and a noticeable reduction in brain size compared to the vehicle, sham group, AMG200 *per se* group. In addition, relative to AMG100 mg/kg andAMG200 mg/kg, there is a significant improvement in the gross-pathology of the brain (Figure 9).

### 3.9. Determination of the Demyelination Volume in PPA-Treated Autistic Rats

**Figure 10:**
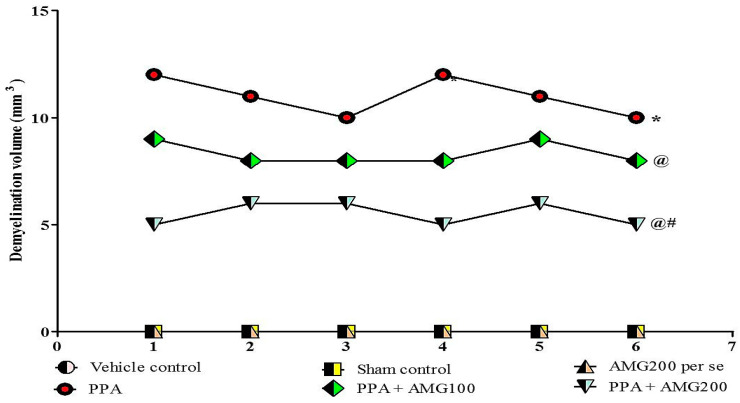
Effect of AMG on demyelination volume in PPA-treated autistic rats.Statistical analysis followed by one-way ANOVA (post-hoc Tukey’s test), * *p* < 0.05 v/s vehicle control; sham control and AMG200 *per se*; @ *p* < 0.05 v/s PPA; @# *p* < 0.05 v/s PPA + AMG100; (*n* = 6 rats per group).

Normal and vehicle control groups showed no significant effect on the size of the demyelination area compared to AMG200 mg/kg *per se*. However, long-term administration of PPA-neurotoxin substantially increased the area of demyelination compared to normal vehicle and AMG200 mg/kg *per se*. There was no change in long-term treatment with AMG200 mg/kg *per se* compared with normal rats. Treatment with AMG100 and 200 mg/kg significantly reduced the demyelination region relative to those groups in which only PPA was used. [ANOVA one-way: F(5,25) = 1.000, *p* = 0.4381] (Figure 10).

## 4. Discussion

Developmental abnormalities due to abnormal brain patterns, circuitry, and cytoarchitecture are related to intellectual disability, including ASD [87,88]. ERK is essential for normal cortical development and functioning and is genetically linked to intellectual disability, including cognitive disorders [89]. Abnormal proliferation of progenitor cells during neurogenesis is the result of hyperactivation of ERK signaling [90]. Several studies have shown the neuroprotective action of AMG in the treatment of neuroinflammation [91], AD [92], amyloid aggregation [93]. The present research investigates the neuroprotective role of AMG against the autistic model of rats induced by PPA. The repeated administration of AMG100 mg/kg and AMG200 mg/kg also demonstrated a neuroprotective effect by enhancing the various pathological features associated with PPA.

ICV-induced PPA is one of the most widely used and well-established experimental animal models for autism. This pre-clinical model of autism can induce many forms of behavioral and neurochemical alteration, similar to people with autism [12].

During the 44-day protocol schedule, we investigated the neuroprotective effect of AMG by conducting behavioral parameters and assessing molecular markers, inflammatory cytokines, neurotransmitters, and oxidative stress markers in rat brain homogenate. In bodyweight measurements, there was a gradual reduction in body weight observed during the protocol schedule as compared to the vehicle, sham, and AMG200 per group. Chronic administration of AMG100 mg/kg and AMG200 mg/kg was effective, and the bodyweight was restored on days 23, 33, and 43 with respect to PPA rats.

Some studies indicate that PPA administration may reduce locomotion activity in rodents [11] in our finding that there is no change in locomotionactivity on day 1 among all groups, there is a significant reduction in locomotionactivity in the PPA group at the end of the protocol schedule compared to vehicle control, sham control and AMG200 *per se*. Chronic administration of low-dose AMG and high-dose AMG was significant, and the locomotionactivity was restored.

Several studies have shown that PPA is responsible for memory loss [94], as regards the behavioral test, in MWM following chronic PPA administration, a significant increase in ELT, and a decrease in TSTQ with severe memory loss. Treatment with low-dose AMG and high-dose AMG showed a significant decline in ELT and increased TSTQ evidence of improved memory loss.

Muscle coordination is also affected by PPA injection in rodents [95]. In our results, there is a substantial increase in the number of slips during beam-crossing tasks in PPA groups compared to vehicle control, sham control, and AMG200 *per se* group. Chronic low-dose and high-dose AMG administration significantly decrease the number of slips suggested to regain rat muscle control as opposed to PPA at the end of the protocol cycle.

Some studies have shown that PPA causes depression inrodents [96]. Our findings indicate that the PPA group showed a significant increase in immobility time while performing the force swim test compared to the vehicle control, sham control, and the AMG200 *per se* group. At the same time, chronic administration of low-dose and high-dose AMG reduces immobility time compared to the PPA group, which showed anti-depressant action of AMG on rodents. However, chronic administration of AMG has demonstrated significant improvement in PPA induces behavioral changes that may be due to its neuroinflammatory inhibitory activity.

We analyze the effect of AMG on ERK signaling to determine the molecular mechanism. ERK/MAPK is a signaling pathway essential for neurogenesis, pathway dysfunction has resulted in many neurological disorders [97,98], and previous studies have shown that ERK hyperactivation is responsible for developing CNS pathologies in the autoimmune ASD animal model [87,99]. In our findings, ELISA was evaluated for ERK in brain homogenate, and we found a significant increase in ERK in PPA rats. Chronic treatment with AMG100 mg/kg and AMG200 mg/kg reduces and controls the ERK level.

Myelin basic protein (MBP) is the structural protein needed to produce the myelin sheath. We have assumed that this is also the key to understanding progenitor proliferation in neurogenesis, which is also a major pathological hallmark of ASD [100]. In the current study, the level of MBP, as quantified by ELISA, has been shown to increase progressively in PPA rats and to normalize in a dose-dependent manner after chronic treatment with AMG. In addition, apoptotic markers, caspase-3, and Bax (pro-apoptotic) increase significantly, while Bcl-2 (Anti-apoptotic) decreases significantly in PPA rats. After treatment, the expression of all three is reversed.

PPA decreased the levels of dopamine, acetylcholine, and serotonin, while increased the levels of glutamate showing neuronal toxicity. The administration of AMG restores the level of neurotransmitters. PPA raises the level of pro-inflammatory cytokines, such as TNF-alphaand IL-1β, which trigger ASD lesions [101]. PPA groups showed an increased level of pro-inflammatory markers compared to the vehicle, sham, AMG200 *per se* group, and our findings show that chronic administration of AMG100 mg/kg and AMG200 mg/kg significantly and dose dependently reduces both TNF-alphaand IL-1β in brain tissue compared to the PPA group.

Oxidative stress is directly related to neurological disorders, and oxidative stress markers have been influenced by PPA administration [102]. Oxidative stress plays an important role in the progression of autism. PPA rats showed a significant increase in the level of oxidative stress markers (AchE, MDA, LDH, and nitrite) while the level of antioxidants (SOD and GSH) decreased when compared to the vehicle, sham, and AMG200 per se group. Chronic administration of AMG to PPA-induced rats significantly reduced the level of oxidative stress markers and increased antioxidant levels.

In our research, the brain’s morphological structure and the coronal sectioning of the brain have also been studied. PPA rat shows alteration of brain structures, markedly reduced size, and damage area seen compared to vehicle, sham, and AMG200 *per se* groups, which reduces morphological alteration with chronic AMG low dose and high dose. In coronal sectioning of the brain, clearly identified tissue was found relative to the PPA group, where, as in the PPA group, damaged meninges andbrain tissue region were seen as relative to the vehicle, sham, and AMG200 *per se* groups. Chronic administration of AMG100 mg/kg and AMG200 mg/kg indicated a reduction in gross pathological changes and demyelination volume. However, current results are only associations in which the neuroprotective activity of AMG in PPA-induced neurobehavioral, molecular and morphological alterations in Autism-treated rats is primarily examined by the reduction of ERK1/2 levels. A further mechanism of action, such as overexpression or deletion of the ERK1/2, as well as additional molecular evidence, such as immunoblotting and immunohistopathology, and gender studies must be validated.

## 5. Conclusions

In conclusion, we demonstrated that AMG down-regulates the proliferation of progenitor cells and decreases apoptosis in rats treated with ICV-PPA via the ERK pathway. This suggests that ERK can cause neuronal cell growth by inhibiting the apoptotic pathway and enhancing myelin-basic protein level in the rat brain. In addition, AMG enhances anti-inflammatory and anti-oxidant activity by lowering inflammatory cytokines such as TNF-α, IL-1β, and reducing oxidative stress marker production. AMG exhibits an anti-apoptotic effect by lowering the level of caspase-3 and Bax while increasing the level of Bcl-2 (anti-apoptotic marker) by inhibiting the ERK signaling in damaged neuronal cells. In addition to this, morphological and gross pathological study of rat’s brain demonstrates AMG’s neuroprotective action against PPA-induced alteration in the brain. Such promising results suggest that AMG might be a potential approach to disorders relating to neurodevelopmental such as autism. Furthermore, we can compare the AMG as a futuristic pharmacological intervention with other standard drug therapy. Apart from the fact that our study has some limitations, we have not performed any western blot, immunohistopathology studies that should be considered for the future.

## Figures and Tables

**Table 1:** Effects of AMG on extracellular signal-regulated kinases (ERK) and myelin basic protein (MBP) levels in PPA-treated autistic rats.

Groups	ERK (ng/mL)	R-MBP (µg/mL)
Vehicle Control	100.5 ± 0.85	100.3 ± 0.82
Sham Control	100.7 ± 0.58	100.7 ± 0.54
AMG200 *per se*	100.6 ± 0.61	100.7 ± 0.59
PPA	250.7 ± 0.51 *	152.9 ± 0.55 *
PPA + AMG100	210.4 ± 0.53 ^@^	145.5 ± 0.60 ^@^
PPA + AMG200	190 ± 0.59 ^@#^	130.7 ± 0.59 ^@#^

Statistical analysis followed by one-way ANOVA (post-hoc Tukey’s test), * *p* < 0.05 v/s vehicle control; sham control and AMG200 *per se*; ^@^ *p* < 0.05 v/s PPA; ^@#^ *p* < 0.05 v/s PPA + AMG100; (*n* = 6 rats per group).

**Table 2:** Effects of AMG on caspase-3, Bax and Bcl-2 level in PPA-treated autistic rats.

Groups	Caspase-3 (ng/mL)	Bax (ng/mg Protein)	Bcl-2 (ng/mg Protein)
Vehicle Control	100.32 ± 0.52	4.80 ± 0.25	28.19 ± 0.25
Sham Control	100.85 ± 0.54	4.80 ± 0.25	28.21 ± 0.26
AMG200 *per se*	100.50 ± 0.52	4.80 ± 0.25	28.20 ± 0.26
PPA	150.93 ± 0.91 *	10.73 ± 0.25 *	18.67 ± 0.56 *
PPA + AMG100	140.92 ± 0.56 ^@^	8.70 ± 0.23 ^@^	21.16 ± 0.58 ^@^
PPA + AMG200	130.48 ± 0.55 ^@#^	6.92 ± 0.25 ^@#^	23.67 ± 0.24 ^@#^

Statistical analysis followed by one-way ANOVA (post-hoc Tukey’s test), * *p* < 0.05 v/s vehicle control; sham control and AMG200 *per se*; ^@^ *p* < 0.05 v/s PPA; ^@#^ *p* < 0.05 v/s PPA + AMG100; (*n* = 6 rats per group).

**Table 3:** Effects of AMG on the evaluation of neurotransmitters level in PPA-treated autistic rats.

	Neurotransmitters Levels			
Groups	5-HT (ng/mg Protein)	Glutamate (ng/mg Protein)	Dopamine (ng/mg Protein)	Ach (ng/mg Protein)
Vehicle Control	36.63 ± 0.55	103.66 ± 0.57	85.48 ± 0.34	9.48 ± 0.26
Sham Control	37.57 ± 0.63	103.52 ± 0.38	87.54 ± 0.54	9.49 ± 0.36
AMG200 *per se*	36.72 ± 0.59	101.52 ± 0.47	85.35 ± 0.60	9.49 ± 0.26
PPA	12.81 ± 0.52 *	278.90 ± 0.58 *	29.36 ± 0.55 *	0.80 ± 0.01 *
PPA + AMG100	15.51 ± 0.60 ^@^	201.82 ± 0.60 ^@^	44.48 ± 0.52 ^@^	5.71 ± 0.61 ^@^
PPA + AMG200	18.29 ± 0.59 ^@#^	172.34 ± 0.59 ^@#^	48.85 ± 0.56 ^@#^	7.18 ± 0.37 ^@#^

Statistical analysis followed by one-way ANOVA (post-hoc Tukey’s test), * *p* < 0.05 v/s vehicle control; sham control and AMG200 *per se*; ^@^ *p*<0.05 v/s PPA; ^@#^ *p*<0.05 v/s PPA + AMG100; (*n* = 6 rats per group).

**Table 4:** Effects of AMG on the evaluation of inflammatory cytokines level in PPA-treated autistic rats.

Groups	Neuroinflammatory Markers	
	TNF-α (pg/mg Protein)	IL-1β (pg/mg Protein)
Vehicle Control	28.83 ± 0.84	12.48 ± 0.54
Sham Control	28.93 ± 0.85	12.56 ± 0.79
AMG200 *per se*	28.41 ± 0.57	12.62 ± 0.68
PPA	66.93 ± 0.99 *	25.90 ± 0.37 *
PPA + AMG100	60.29 ± 0.58 ^@^	20.93 ± 0.37 ^@^
PPA + AMG200	48.37 ± 0.50 ^@#^	16.82 ± 0.33 ^@#^

Statistical analysis followed by one-way ANOVA (post-hoc Tukey’s test), * *p* < 0.05 v/s vehicle control; sham control and AMG200 *per se*; ^@^ *p* < 0.05 v/s PPA; ^@#^ *p* < 0.05 v/s PPA + AMG100; (*n* = 6 rats per group).

**Table 5:** Effects of AMG on the evaluation of oxidative stress markers in PPA-treated autistic rats.

Oxidative Stress Markers						
Groups	AchE (µM/mg Protein)	LDH (Unit/mg Protein)	SOD (µM/mg Protein)	MDA (nM/mg Protein)	Nitrite (µM/mg Protein)	GSH (µM/mg Protein)
Vehicle Control	16.30 ± 0.72	103.41 ± 0.88	453.25 ± 0.56	28.69 ± 0.58	4.14 ± 0.43	31.45 ± 0.35
Sham Control	16.92 ± 0.72	104.50 ± 0.97	453.54 ± 0.59	28.91 ± 0.58	4.14 ± 0.36	30.65 ± 0.40
AMG200 *per se*	16.31 ± 0.79	103.89 ± 0.70	453.53 ± 0.34	28.92 ± 0.37	4.15 ± 0.35	31.01 ± 0.40
PPA	48.89 ± 0.85 *	377.45 ± 0.79 *	310.53 ± 0.61 *	66.86 ± 0.55 *	9.92 ± 0.25 *	6.60 ± 0.37 *
PPA + AMG100	35.38 ± 0.62 ^@^	282.72 ± 0.89 ^@^	323.38 ± 0.54 ^@^	60.71 ± 0.69 ^@^	7.42 ± 0.36 ^@^	11.67 ± 0.36 ^@^
PPA + AMG200	28.32 ± 0.59 ^@#^	257.21 ± 0.56 ^@#^	340.25 ± 0.57 ^@#^	53.25 ± 0.58 ^@#^	5.87 ± 0.24 ^@#^	18.84 ± 0.57 ^@#^

Statistical analysis followed by one-way ANOVA (post-hoc Tukey’s test), * *p* < 0.05 v/s vehicle control; sham control and AMG200 *per se*; ^@^ *p* < 0.05 v/s PPA; ^@#^ *p* < 0.05 v/s PPA + AMG100; (*n* = 6 rats per group).

## Data Availability

The original contributions presented in the study related to body weight and raw data included in the Supplementary Material; further inquiries can be directed to the corresponding authors.

## Supplementary Materials

The original research data can be downloaded at: https://www.mdpi.com/2076-3425/11/3/288/s1, File S1: Body weight raw data; File S2: Original scanned register with body weight and protocol schedule.

## Abbreviations and Definitions

Ach	acetylcholine
AChE	acetylcholinesterase
AD	Alzheimer’s disease
ALS	amyotrophic lateral sclerosis
AMG	alpha mangostin
AP	anterior/posterior
ASD	autism spectrum disorder
BCHe	butyrylcholinesterase
DMSO	dimethyl sulfoxide
DV	dorsal/ventral
ERK	extracellular signal-regulated kinases
HD	Huntington’s disease
HPLC	high performance liquid chromatography
ICV	intracerebroventricular
IL-β	interleukin beta
INCO	instruments & chemicals private limited
LDH	lactate dehydrogenase
LTD	long-term depression
LTP	long-term potentiation
MAPK	mitogen-activated protein kinase
MBP	myelin basic protein
MDA	malondialdehyde
ML	medial/lateral
MS	multiple sclerosis
MWM	Morris water maze
NO	nitric oxide
OPA	o-phthalaldehyde
p.o.	oral route
PD	Parkinson’s disease
PPA	propionic acid
SOD	superoxide dismutase
TIFF	tagged image file format
TNF-α	tumor necrosis factor
TSTQ	time spent in the target quadrant
β-ME	β-mercaptoethanol
