# Correction: Tiwari et al. Neuroprotective Effect of α-Mangostin in Ameliorating Propionic Acid-Induced Experimental Model of Autism in Wistar Rats. *Brain Sci.* 2021, *11*, 288

**DOI:** 10.3390/brainsci14100959

**Published:** 2024-09-25

**Authors:** Aarti Tiwari, Rishabh Khera, Saloni Rahi, Sidharth Mehan, Hafiz Antar Makeen, Yahya H. Khormi, Muneeb U Rehman, Andleeb Khan

**Affiliations:** 1 Department of Pharmacology, Neuropharmacology Division, ISF College of Pharmacy, Moga, Punjab 142001, India; aartitiwarildh@gmail.com (A.T.); rishabhkhera61199@gmail.com (R.K.); saloninamta@gmail.com (S.R.); 2 Department of Clinical Pharmacy, College of Pharmacy, Jazan University, Jazan 45142, Saudi Arabia; hafiz@jazanu.edu.sa; 3 Division of Neurosurgery, Department of Surgery, Faculty of Medicine, Jazan University, Jazan 45142, Saudi Arabia; yakhormi@jazanu.edu.sa; 4 Department of Clinical Pharmacy, College of Pharmacy, King Saud University, Riyadh 11451, Saudi Arabia; muneebjh@gmail.com; 5 Department of Pharmacology & Toxicology, College of Pharmacy, Jazan University, Jazan 45142, Saudi Arabia

**Missing Supplementary Materials:**

In the original publication [1], the Supplementary Materials part was not included. The newly added Supplementary Materials part appears below:

Supplementary Materials: The original research data can be downloaded at: https://www.mdpi.com/2076-3425/11/3/288/s1, File S1: Body weight raw data; File S2: Original scanned register with body weight and protocol schedule.

**Data Availability Statement Updated:**

The Updated Data Availability Statement appears below:

The original contributions presented in the study related to body weight and raw data included in the Supplementary Material; further inquiries can be directed to the corresponding authors.

The authors state that the scientific conclusions are unaffected. This correction was approved by the Academic Editor. The original publication has also been updated.
