# Supplementary material for: Neuroprotective Effect of α-Mangostin in Ameliorating Propionic Acid-Induced Experimental Model of Autism in Wistar Rats
Source: Brain Sci. 2021 Feb 25;11(3):288. doi: 10.3390/brainsci11030288 (PMC7996534; doi:10.3390/brainsci11030288)
Supplement: Supplementary file 1 [file brainsci-11-00288-s001.zip › File S2. Original scanned register with body weight and protocol schedule.pdf]

ISSUE & RETURN PAGE

ISSUE

NOTE BOOK NO. 01

ISSUED TO Aarli Tiwari [illegible]

(Name) (Signature)

ISSUE DATE

DEPARTMENT Pharmacology

M. Pharm / Ph.D M. Pharm

PROJECT TITLE Effect of ERK/MAPK inhibitor and mangostin in ICV propionic a induce experimental model of Autism.

SUPERVISOR (S) Dr. Sidharth Mehan

CONTINUED FROM 31st/Jul/019

S. Mehan 02/08/19

NOTE BOOK NO. 01

RETURN

INSPECTED AND COLLECTED BY DATE

PAGE NUMBER OF LAST ENTRY

RETURN DATE

RECEIVED BY

DATE	TABLE OF CONTENTS	PAGE NO.
31/July/19	Animal observation	1
1/Aug/19	Animal observation	2
2/Aug/19	Animal observation	3
3/Aug/19	Animal Behavioral parameters: In house DAY 1 (vehical group)	4-8
4/Aug/19	Behavioral parameters: In house (DAY: 2	4-8
5/Aug/19	Behavioral parameters: In house DAY: 3.	4-8.
6/Aug/019	Animals behavioral parameters: In house DAY: 4	4-8
7/Aug/019	Animals behavioral parameters In house DAY: 5	4-8
8/Aug/019	Animal observation & Behavioral parameter Animal h	9-13
9/Aug/019	Animal observation & Behavioral parameter Animal	9-13
10/Aug/019	Animal behavioral parameters. Animal house Day 3	9-13
11/Aug/019	Animal behavioral parameters: Animal house Day 4	9-13
12/Aug/019	Animal behavioral parameters: Animal house Day 5	9-13
14/Aug/019	Report of Animal house parameters of Animal	14-15
3/sep/019	Stereotaxic perform	16-21
12/sep/19	Biochemical estimations:	
	Theory	22-34
	calculation of chemicals	29-35
	Result table (observation)	36-41
12/Oct/019	Protocole schedule Roadmap	42-44
13/Oct/019	Animal observation & Training periods.	45-48
14/Oct/019	" " " "	45-52
15/Oct/019	" " " "	45-52
16/Oct/019	" " " "	45-52
11/Nov/019 to 27/Dec/019	Group disease + drug high dose ↳ observation + surgery + Roadmap Protocol	53-72
15/Nov/019 to 2/Jan/020	Group: disease (Toxin only) observation + Road map + surgery. Protocol	73-102
28/Dec/019 to 14/feb/020	Group: Disease + drug low dose ↳ observation + surgery + Road map + Protocol	103-130

DATE	TABLE OF CONTENTS	PAGE NO.
2/Jan/20 to 16/feb/20	Group: α-mangostin 200mg/kg (AM4200) Perse	132-154
	Roadmap, protocol, behavioural parameter observation	"
7 Jan - 16 feb 20	Behavioural Parameter,	"
	Body weight	"
	Locomotor activity	
	Beam crossing task	"
	Morris water maze	"
	Force swim test	"
	(Behavioural Parameters completed)	[illegible]
	Gross patho	
	Biochemical estimations	155
	. Oxidative marker : NT cytokines	"
	. NT level . cell/molecular	"
	. Bodyweight	"

TITLE Protocol Schedule Roadmap BOOK NO.
DATE 12/oct/019

From Page No.

DAY	Date	
-4	13/oct/019	Habituation
-3	14/oct/019	Training period (MWM, FST, LA, BBT)
-2	15/oct/019	
-1	16/oct/019	
0	17/oct/019	Rest, surgery (stereotaxic)
0	18/oct/019	
0	19/oct/019	
1	20/oct/019	
2	21/oct/019	
3	22/oct/019	1oul 0.26 M propionic acid
4	23/oct/019	(Toxin) induction through ICV
5	24/oct/019	
6	25/oct/019	
7	26/oct/019	
8	27/oct/019	
9	28/oct/019	
10	29/oct/019	
11	30/oct/019	
12	31/oct/019	α-mangostin p.o
13	1/Nov/019	

To Page No.

RECORDED BY	SUPERVISOR	DATE	WITNESSED BY	DATE
Aarti Tiwari	Dr. Sidharth Mehan			

TITLE ____ BOOK NO. ____ DATE ____

From Page No. ____

Day	Date	
14	2/Nov/019	
15	3/Nov/019	
16	4/Nov/019	
17	5/Nov/019	
18	6/Nov/019	High dose & low dos[illegible]
19	7/Nov/019	of protocol along
20	8/Nov/019	(α-mangostin 100mg/kg &
21	9/Nov/019	200mg/kg p.o)
22	10/Nov/019	
23	11/Nov/019	→ FST, LA, BCT.
24	12/Nov/019	
25	13/Nov/019	
26	14/Nov/019	
27	15/Nov/019	
28	16/Nov/019	
29	17/Nov/019	
30	18/Nov/019	
31	19/Nov/019	
32	20/Nov/019	
33	21/Nov/019	

To Page No. ____

RECORDED BY: Aarti Tiwari SUPERVISOR: Dr. Sidharth Mehan DATE ____ WITNESSED BY ____ DATE ____

TITLE ____ BOOK NO. ____ DATE ____

From Page No. ____

Day	Date	
34	22/Nov/019	
35	23/Nov/019	
36	24/Nov/019	High & Low dose of
37	25/Nov/019	protocol drug induction.
38	26/Nov/019	
39	27/Nov/019	
40	28/Nov/019	
41	29/Nov/019	Morris water maze
42	30/Nov/019	
43	1/Dec/019	FST, LA, BCT,
44	2/Dec/019	
45	3/Dec/019	In brain homogenate biochemical
46	4/Dec/019	estimations, ERK/MAPK, Rat myelin
47	5/Dec/019	Basic protein, DA, Ach, TNFα, IL-1β, OS.
48	6/Dec/019	morphological sectioning,
49	7/Dec/019	Histopathological
50	8/Dec/019	

To Page No. ____

RECORDED BY	SUPERVISOR	DATE	WITNESSED BY	DATE
Aarti Tiwari	Dr. Sidharth Mehan			

TITLE New schedule BOOK NO.

For rat surgery : Group: Disease + Drug (High dose) DATE 11/Nov/019

From Page No.

Disease + Drug (H.D) (200mg/kg)

Date	Day	Task	Parameters
7/Nov/19	-1	Habituation & training period	Beam walking Task (BCT)
8/Nov/19	-2		Morris water maze (MWM)
9/Nov/19	-3		Locomotor activity (LMA)
10/Nov/19	-4		Force swim test (FST)
11/Nov/19	0	"0 days"	surgery
12/Nov/19	0		Relaxation
13/Nov/19	0		Relaxation
14/Nov/19	1	10 ul given (Toxin-PPA) (ICV)	BW, LCT, BCT
15/Nov/19	2	"	
16/Nov/19	3	"	
17/Nov/019	4	"	
18/Nov/19	5	"	
19/Nov/19	6	"	
20/Nov/19	7		
21/Nov/19	8		
22/Nov/19	9		
23/Nov/19	10		
24/Nov/19	11		
25/Nov/19	12	Treatment α-mangostin 200mg/kg	
26/Nov/19	13		BW, LCA, BCT, FST

To Page No.

RECORDED BY	SUPERVISOR	DATE	WITNESSED BY	DATE
Aadi Thiruni	Dr. Siddharth Mehan		[illegible]	

From Page No. ______

27/Nov/019	14	"	
28/Nov/019	15	"	
29/Nov/019	16	"	
30/Nov/019	17	"	
1/Dec/019	18	"	
2/Dec/019	19	"	
3/Dec/019	20	"	
4/Dec/019	21	"	
5/Dec/019	22	"	
6/Dec/019	23	"	BW, LMA, BCT, FST
7/Dec/019	24	"	
8/Dec/019	25	"	
9/Dec/019	26	"	
10/Dec/019	27	"	
11/Dec/019	28	"	
12/Dec/019	29	"	
13/Dec/019	30	"	
14/Dec/019	31	"	
15/Dec/019	32	"	
16/Dec/019	33	"	Body wt, LMA, BCT, FST
17/Dec/019	34	"	

To Page No. ______

RECORDED BY	SUPERVISOR	DATE	WITNESSED BY	DATE
Aarti Tiwari	Dr. Siddharth Mehan			

TITLE ____________ BOOK NO. ____________ DATE ____________

From Page No. ____________

18/Dec/019	35	"	
19/Dec/019	36	"	
20/Dec/019	37	"	
21/Dec/019	38	"	
22/Dec/019	39	"	
23/Dec/019	40	"	MWM
24/Dec/019	41	"	MWM
25/Dec/019	42	"	MWM, BW, LCA, BCT, FST
26/Dec/019	43	"	MWM, BW, LCA, BCT
27/Dec/019	44	"	MWM (TSTQ)
28/Dec/019	45	sacrifice	Biochemical estimations
29/Dec/019	46		
30/Dec/019	47		
31/Dec/019	48	Brain Isolation	Histo/morphology
1/JAN/020	49		
2/JAN/020	50		

To Page No. ____________

RECORDED BY	SUPERVISOR	DATE	WITNESSED BY	DATE
Aarti Tiwari	Dr. Siddharth Mehan		[illegible]	

TITLE Tabular observations :- BOOK NO. Page No. 61

Body wt: (Disease + high dose) DATE 14 Nov - 26-Dec/19

From Page No.

Group α-mangostine

Body wt:

S.No.	Rat no.	Body wt (g)	Date
1	Rat1	212 g	14/Nov/019
	Rat2	217g	14/Nov/019
	3	213	"
	4	211	"
	5	212	"
	6	214	"
2	1	195	29/Nov/019
	2	199	"
	3	196	"
	4	198	"
	5	200	"
	6	199	"
3	1	193	6/Dec/019
	2	198	6/Dec/019
	3	193	
	4	195	

To Page No.

RECORDED BY	SUPERVISOR	DATE	WITNESSED BY	DATE
Anil Tiwari	Dr. Siddharth Mehan			

TITLE ______ BOOK NO. ______

DATE ______

Page 62

From Page No. ______

S.No.	Rat no.	Body wt.	Date
	5	193	
	6	195	
4	1	210	16/Dec/019
	2	217	16/Dec/019
	3	214	"
	4	210	"
	5	213	"
	6	215	"
5	1	225	26/Dec/19
	2	226	26/Dec/19
	3	221	26/Dec/19
	4	226	"
	5	223"	"
	6	228	"

To Page No. ______

RECORDED BY	SUPERVISOR	DATE	WITNESSED BY	DATE
Aniti Tiwari	Dr. Sidharth Mehan			

TITLE Group 'Disease (Toxin only) BOOK NO. ______ Page No. 73

DATE 15/Nov/[illegible]

Disease group (Toxin only PPA ICV. 26hd 10ul/[illegible]

From Page No. ______

Road map

Date	Day	Tasks	Parameters
16/Nov/019	-4	Habituation	MWM, BW
17/Nov/019	-3		BCT
18/Nov/019	-2		FST
19/Nov/019	-1		LMA
20/Nov/019	Day 1 Toxin gi	surgery	
21/Nov/019	2	Relaxation.	
22/Nov/019	3		
23/Nov/019	4	Toxin (PPA (10ul-ICV)	BW, LCM, BCT
24/Nov/019	5	"	
25/Nov/019	6	"	
26/Nov/019	7	"	
27/Nov/019	8	"	
28/Nov/019	9	"	
29/Nov/019	10	"	
30/Nov/019	11	"	
1/Dec/019	12	"	
2/Dec/019	13	"	
3/Dec/019	14	"	
4/Dec/019	15		
5/Dec/019	16		BW, BCT, LMA, FST

To Page No. ______

RECORDED BY	SUPERVISOR	DATE	WITNESSED BY	DATE
Aarti Tiwari	Dr. Sidharth Mehan		[illegible]	

TITLE ______ BOOK NO. ______

DATE ______

From Page No. ______

Date	Day	Task	Parameters
6/Dec/019	17		
7/Dec/019	18		
8/Dec/019	19		
9/Dec/019	20		
10/Dec/019	21		
11/Dec/019	22		
12/Dec/019	23		BW, BCT, ICM [illegible]
13/Dec/019	24		
14/Dec/019	25		
15/Dec/019	26		
16/Dec/019	27		
17/Dec/019	28		
18/Dec/019	29		
19/Dec/019	30		
20/Dec/019	31		
21/Dec/019	32		
22/Dec/019	33		
23/Dec/019	34		
24/Dec/019	35		
25/Dec/019	36		

To Page No. ______

RECORDED BY	SUPERVISOR	DATE	WITNESSED BY	DATE
Aarti Tiwari	Dr. Siddharth [illegible]			

TITLE	BOOK NO.	Page No.
	DATE	75

From Page No.

Date	Day	Task	Parameter
26/DEC/2019	37		
27/DEC/2019	38		
28/DEC/2019	39		
29/DEC/2019	40		MWM,
30/DEC/2019	41		MWM
31/DEC/2019	42		" MWM "
1/JAN/2020	43		EL, MWM
2/JAN/2020	44		TST0, "
3/JAN/2020	45	sacrifice	Biochemical estimation
4/JAN/2020	46		
5/JAN/2020	47		
6/JAN/2020	48		histo & morpho
7/JAN/2020	49		
8/JAN/2020	50		

To Page No.

RECORDED BY	SUPERVISOR	DATE	WITNESSED BY	DATE
Aarti Tiwari	Dr. Siddhartha [illegible]			

TITLE Tabulated observation BOOK NO.

Group: disease (Toxin only) DATE 23/Nov – 1/Jan/2020

From Page No.

(1) Body wt.

Sr.NO.	Rat NO	Rat body wt (g)	Date
1	1	216	23/Nov/019
2	2	214	" "
	3	210	"
	4	212	"
	5	212	"
	6	211	"
2	1	202	3/Dec/019
	2	200	3/Dec/019
	3	198	"
	4	200	"
	5	197	"
	6	200	"
3	1	180	12/Dec/019
	2	179	12/Dec/019
	3	175	"
	4	175	"
	5	179	"

To Page No.

RECORDED BY SUPERVISOR DATE WITNESSED BY DATE

Aarti Tiwari Dr. Siddharth Mehan

TITLE Body weight. BOOK NO. DATE

From Page No.

Sr. No.	Rat No.	Rat body wt. (g)	Date	
	6	179	12/Dec/019	
4	1	168	22/Dec/19	
	2	162	22/Dec/19	
	3	160	"	
	4	159	"	
	5	161	"	
	6	155	"	
5	1	149	1/Jan/020	
	2	148	1/Jan/020	
	3	149	"	
	4	140	"	
	5	145	"	
	6	140	"	

To Page No.

RECORDED BY	SUPERVISOR	DATE	WITNESSED BY	DATE
Arvind Tiwari	Dr. Siddharth Mehan		[illegible]	[illegible]

TITLE Tabulated observation BOOK NO.

Group: Disease + Low dose DATE 28/Dec/019

Disease + Drug (low dose) 10 mg/kg α-mangostin.

From Page No.

Day	Date	Task	Parameters
28/Dec/019	-4	Habituation	MWM, BW/BCT, FST, LMA
29/Dec/19	-3		BCT, BW, MWM, LMA
30/Dec/19	-2		FST " "
31/Dec/19	-1		LMA " "
1/Jan/020	0	Surgery day.	
2/Jan/020	1	10 ul given (Toxin-PPA) ICV	BW, LCT, BCT
3/Jan/020	2		
4/Jan/020	3		
5/Jan/020	4		
6/Jan/020	5		
7/Jan/020	6		
8/Jan/020	7		
9/Jan/020	8		
10/Jan/020	9		
11/Jan/20	10		
12/Jan/20	11		
13/Jan/20	12	Treatment	
14/Jan/20	13	α-mangostin	BW, LCA, BCT, FST
15/Jan/20	14		
16/Jan/20	15		

To Page No.

RECORDED BY	SUPERVISOR	DATE	WITNESSED BY	DATE
Aarti Tiwari	Dr. Siddharth Mehan			

TITLE ____________________ BOOK NO. ____________

DATE ____________

Page No. 104

From Page No. ____________

Day	Date	Task	Parameter
17/Jan/020	16		
18/Jan/020	17		
19/Jan/020	18		
20/Jan/20	19		
21/Jan/20	20		
22/Jan/20	21		
23/Jan/20	22		
24/Jan/20	23		BW, LMA, BCT, EST
25/Jan/020	24		
26/Jan/020	25		
27/Jan/20	26		
28/Jan/20	27		
29/Jan/20	28		
30/Jan/20	29		
31/Jan/20	30		
1/Feb/20	31		
2/Feb/20	32		
3/Feb/20	33		BW.
4/Feb/20	34		
5/Feb/20	35		

To Page No. ____________

RECORDED BY	SUPERVISOR	DATE	WITNESSED BY	DATE
Aarti Tiwari	Dr. S. Menon			

TITLE ______ BOOK NO. ______ DATE ______

Page No. 105

From Page No. ______

Day	Date	Task	Parameter
6/feb/20	36		
7/feb/20	37		
8/feb/20	38		
9/feb/20	39		
10/feb/20	40		MWM (EC)
11/feb/20	41		"
12/feb/20	42		"
13/feb/20	43		BCT, FST, LA, MWM
14/feb/20	44		(TST) ~~FST, LA, BCT~~
15/feb/20	45	sacrifice	Biochemical
16/feb/20	46		estimation
17/feb/20	47		
18/feb/20	48		Histo &
19/feb/20	49		morpho
20/feb/20	50		

To Page No. ______

RECORDED BY	SUPERVISOR	DATE	WITNESSED BY	DATE
Aarti Tiwari	Dr. S. Mohan			

TITLE Tabulated observation BOOK NO.
Group: Disease + Drug (Low dose) DATE

From Page No.

Body Weight (g)

S.NO	Rat NO	Body weight	Date
1	1	216	2/Jan/2020
	2	210	2/Jan/2020
	3	212	
	4	211	
	5	212	
	6	214	
2	1	198	14/Jan/2020
	2	195	14/Jan/20
	3	199	
	4	196	
	5	199	
	6	200	
3	1	205	24/Jan/2020
	2	199	24/Jan/2020
	3	203	
	4	201	
	5	202	

To Page No.

RECORDED BY Aarti Tiwari SUPERVISOR Dr. Siddharth Mehan DATE WITNESSED BY DATE

TITLE ______ BOOK NO. ______ DATE ______

Page No. 121

From Page No. ______

S.NO	Rat NO	Body weight	Date
	6	204	
4	1	210	3/feb/20
	2	205	3/feb/2020
	3	206	
	4	205	
	5	207	
	6	208	
5	1	212	13/feb/2020
	2	208	13/feb/2020
	3	210	
	4	210	
	5	210	
	6	211	

To Page No. ______

RECORDED BY Aastha Tiwari SUPERVISOR Dr. Siddharth Mohan DATE

WITNESSED BY DATE

TITLE Pense group
HCT Road map.

BOOK NO.

DATE

From Page No.

Days	Date	Task	Parameters
-4	2/Jan/2020	Habituation +	BW, MWM, FST.
-3	3/Jan/2020	Training period	IMA. BCT.
-2	4/Jan/2020		"
-1	5/Jan/2020		"
0	6/Jan/2020	Resting period Rest	
1	7/Jan/2020		BW, FST, BCT, IMA.
2	8/Jan/2020		
3	9/Jan/2020		
4	10/Jan/2020		
5	11/Jan/2020		
6	12/Jan/2020		
7	13/Jan/2020		
8	14/Jan/2020		
9	15/Jan/2020		
10	16/Jan/2020		
11	17/Jan/2020		
12	18/Jan/2020	d-mangostin [illegible]mg/kg	
13	19/Jan/2020		BW, LCM, BCT, FST
14	20/Jan/2020		

To Page No.

RECORDED BY	SUPERVISOR	DATE	WITNESSED BY	DATE
Aidi Thakur	Dr. Siddharth Mehan			

TITLE ____________ BOOK NO. ____________

DATE ____________

From Page No. ____________

Day	Date	Task	Parameter
15	21/Jan/020		
16	22/Jan/020	"	
17	23/Jan/020	"	BW, FST, LMA, BCT
18	24/Jan/020	"	
19	25/Jan/020	"	
20	26/Jan/020	"	
21	27/Jan/020	"	
22	28/Jan/020	"	
23	29/Jan/020	"	
24	30/Jan/020	"	
25	31/Jan/020	"	
26	1/feb/020	"	
27	2/feb/020	"	
28	3/feb/020	"	
29	4/feb/020	"	
30	5/feb/020	"	
31	6/feb/020	"	
32	7/feb/020	"	
33	8/feb/020	"	BW

To Page No. ____________

RECORDED BY: Aarti Tiwari — SUPERVISOR: Dr. Siddharth Mohan — DATE ____________

WITNESSED BY ____________ DATE ____________

TLE ____________________ BOOK NO. ____________ Page No. 135

DATE ____________

From Page No. ________

Day	Date	Task	Parameter
34	9/feb/020	"	
35	10/feb/020	"	
36	11/feb/020	"	
37	12/feb/020	"	
38	13/feb/020	"	
39	14/feb/020	"	
40	15/feb/020	"	EL (MWM)
41	16/feb/020	"	EL "
42	17/feb/020	"	"
43	18/feb/020	"	", RWFST, BCT, LMA
44	19/feb/020	"	TSTQ.

To Page No. ________

RECORDED BY	SUPERVISOR	DATE	WITNESSED BY	DATE
[illegible] Tiwari	Dr. Siddharth Mehan			

TITLE Tabular observation BOOK NO.

Group: AMG 200 per se DATE

From Page No.

Body weight.

S.NO	Rat no.	Rat weight(g)	Date
1	1	212	7/Jan/020
	2	214	
	3	210	
	4	216	
	5	212	
	6	212	
7	1	217	19/Jan/020
	2	221	
	3	219	
	4	224	
	5	219	
	6	218	
13	1	227	29/Jan/020
14	2	230	
15	3	227	
16	4	231	
17	5	229	
18	6	230	

To Page No.

RECORDED BY	SUPERVISOR	DATE	WITNESSED BY	DATE
Aarti Tiwari	Dr. Sidharth Mehan			

TLE ____ BOOK NO. ____

DATE ____

om Page No. ____

Body weight

S.NO	Rat NO	Rat weight (g)	Date	
4	1	238	8/feb/20	
	2	240		
	3	240		
	4	240		
	5	239		
	6	245		
5	1	254	18/feb/20	
	2	258	16/feb/020	
	3	260		
	4	257		
	5	256		
	6	259		

To Page No. ____

RECORDED BY SUPERVISOR DATE WITNESSED BY DATE

Aarti Tiwari Dr. Sidharth Mehan
